# A High-Aspect-Ratio Deterministic Lateral Displacement Array for High-Throughput Fractionation

**DOI:** 10.3390/mi15060802

**Published:** 2024-06-18

**Authors:** Jonathan Kottmeier, Maike S. Wullenweber, Ingo Kampen, Arno Kwade, Andreas Dietzel

**Affiliations:** 1 Institute of Microtechnology, TU Braunschweig, 38124 Braunschweig, Germany; 2 Center of Pharmaceutical Engineering (PVZ), TU Braunschweig, 38106 Braunschweig, Germanya.kwade@tu-braunschweig.de (A.K.); 3 Institute for Particle Technology, TU Braunschweig, 38104 Braunschweig, Germany

**Keywords:** deterministic lateral displacement (DLD), microfluidics, high throughput, high aspect ratio, size-dependent fractionation

## Abstract

Future industrial applications of microparticle fractionation with deterministic lateral displacement (DLD) devices are hindered by exceedingly low throughput rates. To enable the necessary high-volume flows, high flow velocities as well as high aspect ratios in DLD devices have to be investigated. However, no experimental studies have yet been conducted on the fractionation of bi-disperse suspensions containing particles below 10 µm with DLD at a Reynolds number (Re) above 60. Furthermore, devices with an aspect ratio of more than 4:1, which require advanced microfabrication, are not known in the DLD literature. Therefore, we developed a suitable process with deep reactive ion etching of silicon and anodic bonding of a glass lid to create pressure-resistant arrays. With a depth of 120 µm and a gap of 23 µm between posts, a high aspect ratio of 6:1 was realized, and devices were investigated using simulations and fractionation experiments. With the two-segmented array of 3° and 7° row shifts, critical diameters of 8 µm and 12 µm were calculated for low Re conditions, but it was already known that vortices behind the posts can shift these values to lower critical diameters. Suspensions with polystyrene particles in different combinations were injected with an overall flow rate of up to 15 mL/min, corresponding to Re values of up to 90. Suspensions containing particle combinations of 2 µm with 10 µm as well as 5 µm with 10 µm were successfully fractionated, even at the highest flow rate. Under these conditions, a slight widening of the displacement position was observed, but there was no further reduction in the critical size as it was for Re = 60. With an unprecedented fractionation throughput of nearly 1 L per hour, entirely new applications are being developed for chemical, pharmaceutical, and recycling technologies.

## 1. Introduction

In many industrial fields, particles with well-defined sizes are needed. To generate well-defined particle suspensions, different microfluidic methods relying on fluidic effects for particle fractionation have been developed, including pinched flow fractionation, spiral-channel fractionation, multi-orifice flow fractionation (MOFF), and deterministic lateral displacement (DLD) [1]. In addition, there are methods available for particle separation that rely on external forces such as dielectrophoresis and magnetophoresis [1]. Most of these methods are designed for low Reynolds (Re) numbers and use viscous forces or combinations of viscous and actively induced external forces [1]. However, some methods such as MOFF and fractionation in spiral channels are based on inertial forces that only come into play above Re = 1, requiring typical flow rates in the range of a few mL/min [1,2]. In addition, DLD has been extensively studied at higher Reynolds numbers, although it was originally designed to operate at Stokes flow conditions (Re << 1) [3,4]. A DLD array consists of an inclined array of microposts, with a critical diameter (*D_c_*) for fractionation, which is defined by the geometry [3,4]. By bumping on the posts, particles larger than *D_c_* are displaced laterally. Smaller particles remain on the streamline and are not displaced, following a zigzag motion. *D_c_* is defined by the array geometry according to

(1) $$D_{c}=1.4\;g\;{tan\;(\theta )}^{0.48}$$

where *g* is the gap between posts and *θ* is the tilt angle, which is determined by the lateral shift in the subsequent post positions [3,4,5]. The derivation of *D_c_* based on the width of the first streamline can be found in the literature [5,6]. In addition to zigzag and displacement, another mode of motion has been described in the literature, the so-called zig-bump or mixed mode, a motion of particles that are partially displaced. This means that the particles are displaced but not at every post, resulting in a broadening of the final positioning of the particles [7]. This type of motion is created by an asymmetric distribution of flow lane widths between adjacent posts, meaning that the first streamline is at a different width (perpendicular to the flow) than the last streamline [7]. At higher Reynolds numbers, up to Re = 100, simulative studies have shown increasing flow anisotropy, which causes the average flow direction to not be aligned with the direction of the applied pressure gradient, leading to a shift in the position of the maximal velocity between adjacent posts [8]. The anisotropy of the flow profile in the investigated array was based on an imbalance of the lift and drag forces, which are induced by the inertial flow above Re = 1 [8]. Although only fluid flow was investigated in that study, it is expected that this anisotropy will lead to mixed motion. In addition, high concentrations and high throughputs were simulated, taking into account the friction between the posts and the fluid, as well as particle–particle interactions. It has also been described that particle–particle interactions, as well as agglomerations around posts, will lead to mixed motion [9].

A second finding from the simulations is that static vortices form behind the posts at higher Re, leading to a reduced critical diameter [10,11,12]. The shape of the static vortices increased from Re = 5 to Re = 75. It has been postulated that at higher Re (Re > 75), the vortices are fully developed and no longer influence the critical diameter [4,13]. Based on these simulation data, Aghilinejad et al. [13] introduced an alteration of Equation (1) that not only accounts for the geometry but also the Reynolds number Re, given as Equation (2).

(2) $$D_{c}=1.4\;g\;{\tan\left(\theta \right)}^{0.48}\;e^{-0.01Re}$$

The first experiments at higher Re and increased flow rates were carried out with an upscaled DLD model designed for particles of about 100 µm in diameter [10,12]. With tracer particles that were small in comparison to the critical diameter, the static vortices behind circular posts could be visualized [10,12]. The first high-throughput applications in miniaturized devices focused on the up-concentration of circulating tumor cells (CTCs) by displacing the bigger tumor cells towards the center of the array [3,4]. Furthermore, triangular posts have been used to increase the throughput to a max. of 10 mL/min [14]. With circular and triangular pillars, a shift in the displacement was observed at higher Reynolds numbers, caused by the formation of static vortices behind the posts [10,11,12]. To reduce the static vortices, airfoil-shaped posts have also been investigated [14]. The effects at higher Reynolds numbers and their influence on *D_c_* were studied in simulations and experiments [8,9,10,11,12,13,14,15,16,17].

Equations (1) and (2) are based on simulations assuming an infinite height of the posts and have been shown to be applicable to experiments with high-aspect-ratio systems [18]. For a low aspect ratio, Equations (1) and (2) do not account for the more complex 3D flow between adjacent posts [18]. The Reynolds number *Re* in Equation (2) is calculated using the dynamic viscosity (*η*) and density (*ρ*) of water (since an aqueous buffer is taken), the velocity between adjacent posts (*v*), and the characteristic dimension (*l_hyd_*), which has been used to represent the gap between posts in the DLD literature to date. Although Equation (2) has been shown to be applicable for a Re of up to 75 in simulations of the particle trajectory and flow field, it is not suitable for predicting the critical diameter for even higher values of Re since it is known that the vortices behind the posts do not further evolve [4,13]. However, there has been no experimental investigation in this high-Re regime.

Two main manufacturing processes for DLD devices have been described in the literature, namely, polydimethylsiloxane (PDMS) softlithography and silicon microfabrication [3,4]. PDMS has the inherent advantage of being cheaper and easier to manufacture compared to silicon [3,4]. However, DMS posts cannot be designed with very small diameters as this would compromise the stability of the DLD array [19]. In addition, PDMS deforms under pressure, making fractionation problematic at higher flow rates [19]. Using the more expensive and time-consuming silicon microfabrication technology in combination with anodically bonded glass, pressure-stable systems that are not prone to deformation can be realized [3,4,16]. Another key advantage of silicon microfabrication technology is the ability to produce posts with a much higher aspect ratio than 4:1, which is limited by the capabilities of dry etching, including the photolithography technology and the stability of the silicon posts, with particular attention to the sidewall angle. Consequently, due to the stability of the posts in a high-pressure application, the aspect ratio cannot be infinitely large. If it is subjected to the same pressure, a deeper channel will allow for a higher volumetric flow rate compared to a shallower channel. The largest reported channel depth (array height) in miniaturized DLD devices was 160 µm [20]. These arrays with 40 µm gaps, corresponding to an aspect ratio of 4:1, were used to concentrate and separate cancer cells in the center of the array [14,20]. In other publications, the gap aspect ratio has been reported to be between 2:1 and 3:1 [3,4]. To date, a flow rate of 14 mL/min, resulting in Re = 58, has only been achieved in a DLD device with circular posts at a gap size of 50 µm to address particles in a range of 10 to 20 µm in a non-binary suspension containing only one particle size [11]. No experimental investigations of binary particle fractionation have been conducted at Reynolds numbers above 60 with DLD devices that have aspect ratios greater than 4:1 and particles smaller than 10 µm. In order to investigate fractionation using DLD at even higher Reynolds numbers and considerably increased throughput, we used suitable dry etching processes to manufacture arrays with a height of 120 µm, resulting in an aspect ratio of 6:1, and investigated these using simulations and experiments.

## 2. Materials and Methods

### 2.1. Concept of the Segmented DLD

**Figure 1:**
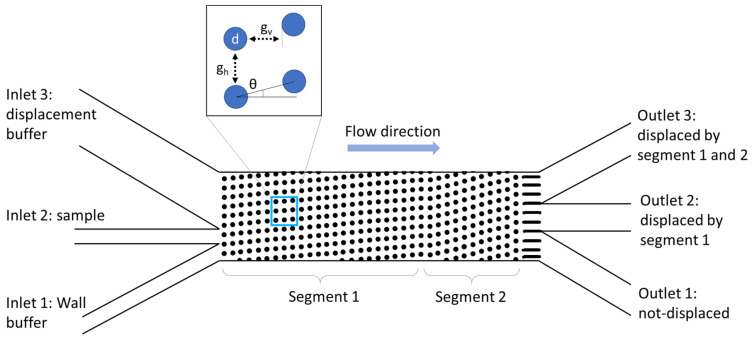
Schematic illustration of a two-segmented DLD post array for particle fractionation. In the detailed view, the horizontal gap (*g_h_*), vertical gap (*g_v_*), post diameter (*d*), and tilt angle (*θ*) are illustrated. In the investigated array, *g_h_* and *g_v_* were identical and thus labeled as *g*.

To limit the fluidic resistance and to ensure high throughput, the array used in this study consisted of two displacement segments, resulting in three fractions, as illustrated in Figure 1. The two segments were arranged in a chirped mode to have an identical gap of 23 µm and post diameter of 17 µm but different tilt angles (*θ*) of 2.7° and 7.1°, resulting in a periodicity of *N* = 20 and *N* = 8 respectively. This setup allowed for three different outlet fractions, as depicted in Figure 1: the first for non-displaced particles, the second for particles exceeding a critical diameter *D_c_* = 8 µm in the first segment, and the third for particles exceeding *D_c_* = 11 µm, as calculated for low Re numbers according to Equation (1). As in our previous work, the DLD system consisted of three inlets to prevent the particles from reaching the sidewall [16]. The volumetric flow rates through the inlets are shown in Table 1 and were chosen to obtain homogenized flow velocities at the DLD entrance.

### 2.2. Description of the Numerical CFD-DEM Model

The system was simulated in sections, with one period considered for each segment. For the simulation setup, the CFDEM^®^ 3.8.1 coupling software was used to couple computational fluid dynamics (CFDs) and the discrete element method (DEM) [21]. The flow field in the microchannel was simulated using the CFD software OpenFOAM^®^ 5.x, and the particle dynamics were simulated using the DEM software LIGGGHTS 3.8.0. Both processes were coupled in a resolved manner using the immersed boundary method (IBM) [22]. In this publication, the fluid–solid interaction was coupled in a two-way mode to account for both the fluid forces and moments on the particle and the influence of the particle on the fluid in six degrees of freedom (6-DOF). To account for short-range hydrodynamic forces between the solids, which cannot be resolved by the fineness of the finite volume grid, a lubrication correction was implemented on the DEM side. A detailed explanation of all physical principles and equations concerning the processes in the simulations can be found in our previous publication [17].

**Figure 2:**
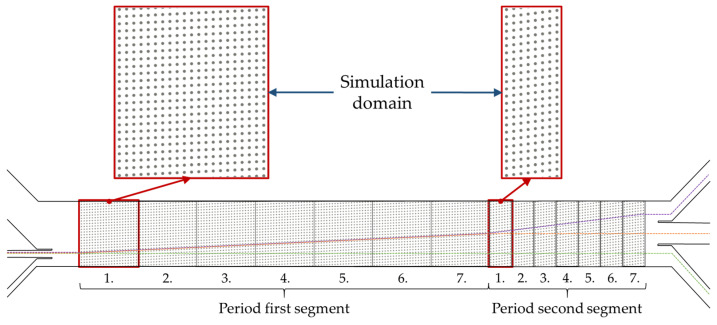
Schematic representation of the two simulation domains and their theoretical position within the microarray. The simulation domains have periodic boundary conditions in the main flow direction. The particle passes through the domain seven times each, allowing the real case to be simulated with reduced computational capacity. The colored lines illustrate the three theoretical cases: zigzag–zigzag (green), displacement–zigzag (orange), and displacement–displacement (purple).

In the simulations, the two segments of the microchannel were considered separately. The width of the simulation domain corresponds to the full width of the channel in order to ensure the same influence of wall effects as in the real case (23 posts as depicted in Figure 2). The length of the domain in the flow direction corresponded to the periodicity *N*, which was 21 post rows in the first segment and 8 post rows in the second segment (according to the relationship $N={\tan\left(\theta \right)}^{-1}$ [3,4,16]). Periodic boundary conditions at the inlet and outlet patches for both fluid and particles allowed the observation of the particle behavior over multiple periods. The inlet velocity of the fluid was controlled by a momentum source, which ensured that the average velocity at the inlet cross-section was a predetermined velocity (see Table 2). As in the real microsystem, the cylindrical posts had a diameter of *d* = 17 µm. The gap between the posts in both lateral and flow directions was *g* = 23 µm. Due to computational constraints, the full depth of 120 µm of the microsystem was not simulated. The depth of the simulation domain and thus, the length of the cylindrical posts, was twice the simulated particle size in order to keep the wall effects on the particle comparable. Slip (Dirichlet) boundary conditions for the fluid velocity were applied to the walls orthogonal to this axis, resulting in a uniform flow profile over the depth, mimicking the condition in the center of a very deep channel. No-slip (Dirichlet) fluid velocity boundary conditions were applied to the lateral side walls and the post walls. For pressure, Neumann boundary conditions were applied to all the walls, except the inlet and outlet walls. The simulation domain was discretized using a block-structured mesh. The mesh fineness corresponded to 21 cells over the length of the gap. In addition, the mesh was dynamically refined by an additional level in the proximity of a particle to achieve the recommended minimum of eight cells per particle diameter [22].

Simulations were performed with a single particle at a time. First, segment 1 was simulated for Re = 18, 60, and 90, respectively, and the particle diameters of 5 µm and 10 µm, respectively, as shown in Table 2. The starting position of the particle for all the simulations of segment 1 was lateral between the 5th and 6th posts of the first row of posts, as shown in Figure 2, corresponding to the experimental position of the particle inlet channel. The particle was then passed through the simulation domain of segment 1 seven times to reproduce the real case. The lateral position after these seven periods was used as the inlet position for the particle into the second segment, where the same procedure was otherwise repeated. The final position after seven passes through each of the two domains thus corresponds to the passage through the entire microsystem and can be compared to the experimental studies. A summary of all the DEM parameters is given in Table 3.

### 2.3. Fabrication of the DLD Arrays

**Figure 3:**
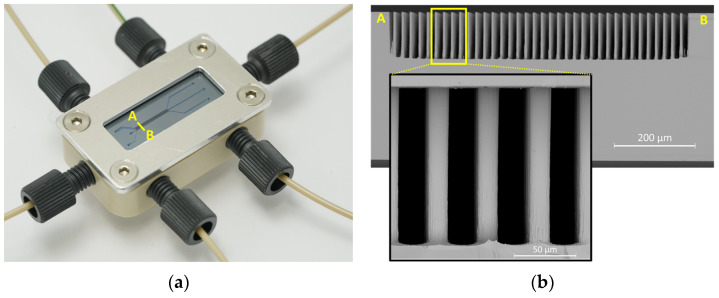
(**a**) Photo of the system mounted on the PEEK holder and fixed with a metal frame. (**b**) SEM images of the array along the line A–B from (**a**) and a detailed zoomed-in view of three posts.

The DLD array patterns were defined using a standard photolithography process on a 4” silicon wafer, consisting of spin-on resist coating and UV exposure through a chrome-coated glass mask. The full details of the photolithography process can be found in Supplementary Material File S1. An Oxford Instruments PlasmaPro100 equipped with a Cobra etch chamber (Oxford Instruments, Oxford, UK) was used for dry etching. To achieve a depth of 120 µm, a 3-step Bosch process was programmed on the instrument, consisting of a deposition (C_4_F_8_) step, a breakthrough (SF_6_) step, and an etch (SF_6_) step. These steps were repeated 150 times resulting in a post structure, as shown in Figure 3b as an overview, with a zoomed-in detailed view of a few posts. The full details of the Bosch process can be found in Supplementary Material File S1. Prior to femtosecond laser ablation of the fluidic connection, the backside was photolithographically patterned with alignment marks for laser positioning. In addition, the array was filled with a tick resist layer Ma-N 1275 (Microchemicals, Berlin, Germany) to prevent dust particles generated during ablation from entering the array. As a final step before bonding, the structured and cleaned wafer was thermally oxidized, resulting in a 300 nm SiO_2_ layer to render the surface hydrophilic and lower the clogging probability. As described in our previous publication, the oxidized wafer was anodically bonded to a glass wafer at a higher voltage (700 V) than required for non-oxidized wafers [16].

### 2.4. Experimental Setup

As illustrated in Figure 1, Section 2.1, the system consisted of three inlets. Inlets 1 and 2 were at identical widths, whereas inlet 3 for the displacement buffer was seven times wider. To achieve throughputs in the mL/min range, a setup of three mid-pressure modules (Nemesys from Cetoni GmbH, Korbussen, Germany) equipped with stainless steel syringes was used. Inlets 1 and 2 were fed by a 10 mL syringe (with a maximal pressure of 50 bar), and the displacement buffer was fed by a 25 mL syringe (with a maximal pressure of 20 bar), allowing a duration of approx. 2 min duration for an experiment with a maximal throughput of 15 mL/min. To investigate the pressure in the inlet channels and syringes, the system was flushed with filtered water at all investigated flow rates, while a pressure monitor (Cetoni GmbH, Korbussen, Germany) was mounted in between the 25 mL syringe and the inlet of the system. A pressure of 15 bar was measured at the maximal flow rate. To connect the syringes to the microfluidic system with a tight pressure, standard high-pressure liquid chromatography (HPLC) connectors and 1/16” PEEK tubes with an internal diameter of 0.75 mm were used. The system was mounted on a CNC-machined PEEK holder with sealing rings and an aluminum lid for fixation, as depicted in Figure 3a. The details of the complete setup, including the syringe pumps and the microscope for observation, can be found in Supplementary Material File S1.

**Figure 4:**
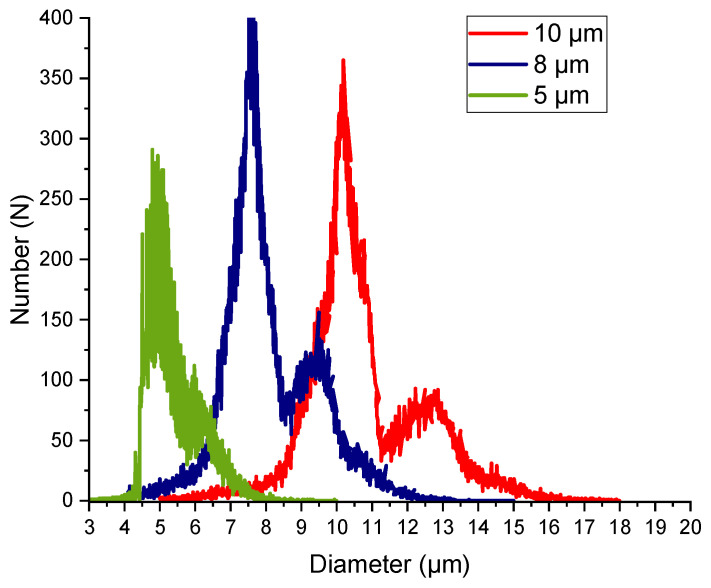
Size distributions measured with Orflo Moxi-Z.

Red fluorescent particles of 5 µm and 10 µm and green fluorescent particles of 8 µm were purchased from Microparticles GmbH (PS FluoRot and PS-FluoGreen, Microparticles GmbH, Berlin, Germany). Green fluorescent particles of 2 µm, 5 µm, and 10 µm were purchased from Distrilab (FluoroMax, Thermo-Fisher, distributed by Distrilab Particle Technology, Leudersend, The Netherlands). The particle size distributions of the particles used were measured using an Orflo Moxi-Z cell counter (Orflo Technologies, Ketchum, ID, USA) after diluting 20 µL of the stock solutions with 1 mL PBS (phosphate buffered saline), resulting in a particle concentration of 0.01% m/V. The results for 5 µm, 8 µm, and 10 µm are depicted in Figure 4. As the smallest detectable particle size was 3 µm, 2 µm particles could not be measured. For the fractionation experiments, binary particle suspensions with two colors were prepared by diluting fluorescent particles at a concentration of 2.5% m/V to a concentration of 0.01% m/V with DI water for each color. No additives such as surfactants were used in these experiments. At the volumetric flow rates given in Table 1, the particle combinations given in Table 4 were investigated.

For observation and video recording, the PEEK holder with the DLD system was placed on a homemade aluminum fixture on the xy-stage of an inverted microscope (Axio Observer, Zeiss, Göttingen, Germany) with darkfield and fluorescence illumination, equipped with a 10× lens and a 1.6× photo adapter. Two sets of filters were available: one for green fluorescent protein (exc. 470 nm/em. 505 nm) and one for rhodamine (exc. 535 nm/em. 605 nm). Two CoolLED lamps at 470 nm and 535 nm were attached to the microscope.

For the video recording, the first white light was used to determine the positions of the outlets, and the system was rinsed with DI water. Then, the binary particle suspension was introduced into the system through the sample inlet. After 1 min, the fluorescence video recording was started.

The desired total flow rate was divided between the inlet channels, taking into account the individual width of each inlet channel. The respective Reynolds numbers were determined as previously described [16]. The velocity (v) was calculated from the channel width by subtracting the sum of all the post diameters; the channel height, and the total volume flow rate. The data for pure water (density *ρ =* 1000 kg/m^3^ and viscosity *η* = 10^−3^ Pa·s) were used, and the characteristic dimension (*l_hyd_*), i.e., the gap between the posts, was used as usually described in the DLD literature [3,4].

### 2.5. Fractionation Profiles from Fluorescence Images

**Figure 5:**
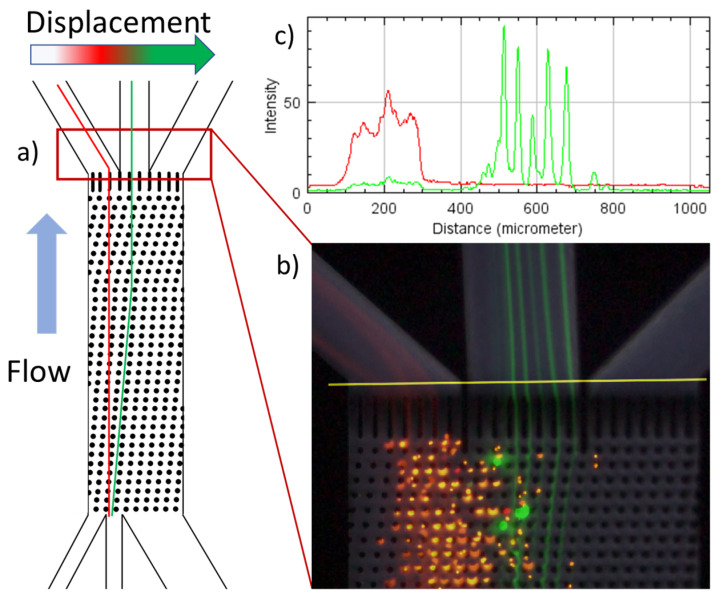
Illustration of the fractionation profile generation based on fluorescent images with the example of PS particles with a diameter of 10 µm (green) and 5 µm (red) at Re = 18. (**a**) Illustration of the positioning of the images within the array fed with a binary particle suspension. (**b**) Image containing a gray-colored background and the particle traces, merged through image editing. The yellow line indicates the positioning of the fractionation profiles. (**c**) Fractionation profile obtained with the RGB Plot function in ImageJ 1.54f.

The videos for data evaluation were generated using a Sony Alpha 7 Mk3 camera (Sony Europe, Berlin, Germany) attached to the microscope with video recording at 100 fps at ISO setting 52000. To open the MP4 videos in ImageJ, the FIJI distribution 1.54f was used in combination with the FFMPEG converter. For each dataset, approximately one minute of video material was generated, containing red and green fluorescence, with at least 10 s for each color. The 10 s of material for each color was then stacked by the average function (z-stacking) in ImageJ. Each of the color images was subsequently used for the designated red and green channels of the final image evaluated, as depicted in Figure 5b. In addition to the fluorescent colors, a gray-colored image of the outlet section was also added for easier visualization. A fractionation profile was generated directly behind the end of the array for all three outlets, as indicated by the yellow line in Figure 5b. To avoid differences due to a small shift in the camera position as well as stuck particles, only a line that was perpendicular to the flow direction was used for evaluation. The profiles were generated with the function “RGB Profile Plot” in ImageJ, as illustrated in Figure 5c. The measured intensities were scaled to obtain values ranging from zero to one.

## 3. Results and Discussion

### 3.1. Simulations

**Figure 6:**
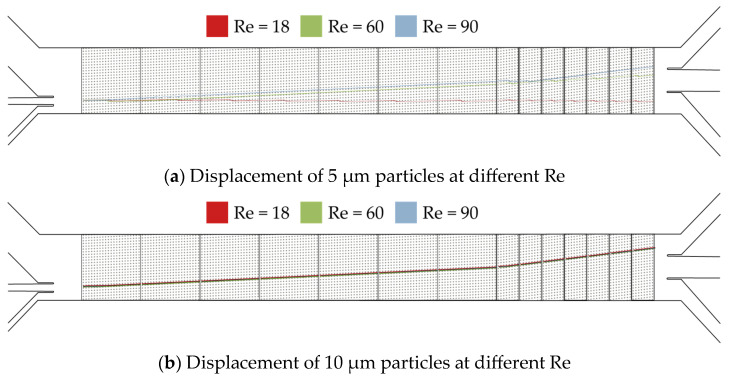
Simulation results of (**a**) the particle trajectory at Re = 18, 60, and 90 for particles with a diameter of 5 µm and (**b**) the particle trajectory at Re = 18, 60, and 90 for particles with a diameter of 10 µm.

**Figure 7:**
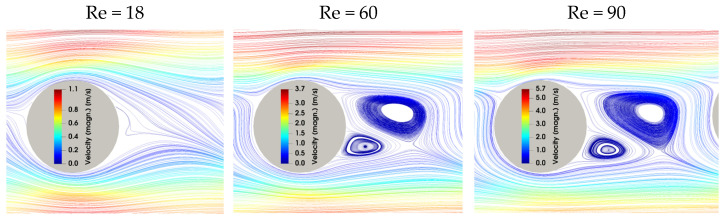
CFD simulations to investigate the behavior of the emerging static vortices behind a micropost. At Re = 18, there is only a small vortex visible. At Re = 60 and Re = 90, there are large vortices behind the posts that are responsible for the shift in critical diameter. The geometrical dimensions of the vortices remain largely unchanged between Re = 60 and Re = 90.

The simulated trajectory of the particles at different Re numbers through the array is depicted in Figure 6. Based on the simulations, it was predicted that the fractionation of particles with sizes of 5 µm and 10 µm would be possible only at Re = 18 and Re = 60. At Re = 90, due to the change in the mixed mode for the 5 µm particles, both sizes were expected to result in the third outlet. Consequently, only a fractionation of 2 µm and 10 µm at Re = 90 was predicted by the simulations. When comparing the 5 µm particles to the 10 µm particles at Re 90, it can be observed that the smaller particles were displaced less far compared to the larger ones. However, the difference in the trajectory of the 5 µm particles was sufficient to change the outlet. Given that the difference in the particle position observed in the simulation was less pronounced than anticipated by Equation (2), we proceeded to simulate the static vortices behind a micropost at Re = 18, 60, and 90, as shown in Figure 7. The shape and geometrical dimensions of the vortices behind a post exhibited minimal variation between Re = 60 and Re = 90, with the exception of an increase in the absolute velocity with Re. This further confirmed the conclusion that Equation (2) is only valid until Re = 75. However, it is possible that the increasing inertial effects influence the particle trajectory.

### 3.2. Experimental Results

**Figure 8:**
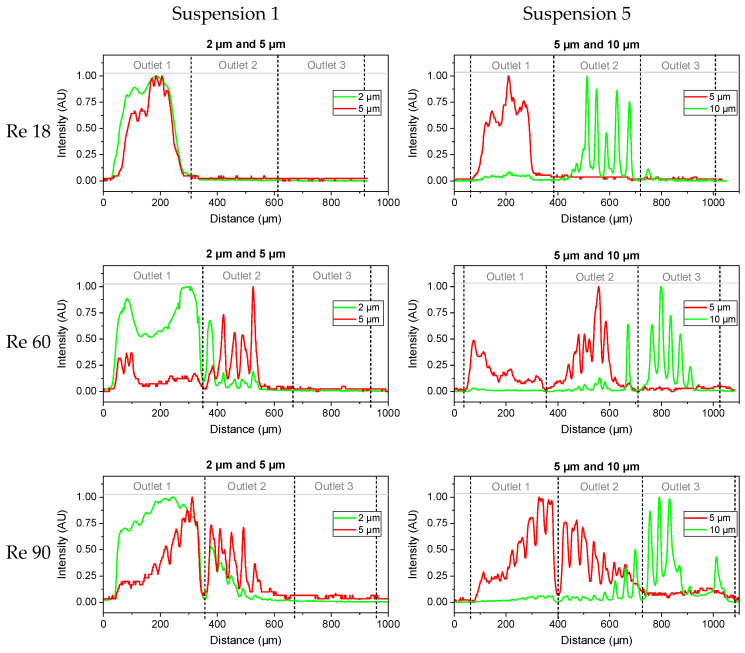
Experimental fractionation profiles obtained with binary suspensions containing 2 µm and 5 µm particles as well as 5 µm and 10 µm particles. From top to bottom, the Reynolds number increases from 18 (3 mL/min) to 60 (10 mL/min) to 90 (15 mL/min). Outlet 1 received non-displaced particles, outlet 2 received particles displaced in the first segment, and outlet 3 particles were displaced in both segments. Since the positioning of the profiles was achieved manually, the positions of the outlets are marked by dashed vertical lines.

For the first time, binary suspensions containing particles smaller than 10 µm were investigated experimentally in a DLD array with circular posts and a gap size smaller than 50 µm above Re = 60. Thus, the outlet region of the array was investigated, as shown in Figure 5. Exemplary fractionation profiles were obtained by the scaled RGB plots of suspensions 1 and 5 (Table 4) with volumetric flow rates according to Table 1, as depicted in Figure 8. The flow rates for Re = 90 in this experiment can result in a velocity between posts of 9 m/s and Re = 208, which can facilitate the comparison to a low-aspect-ratio array of 3:1. To achieve Re = 90 in such an array, an overall flow rate of just 6.4 mL/min would be sufficient. The experiments were conducted at least two times with different arrays from a single fabrication batch. The experiments at Re = 90 were repeated multiple times since the syringes had to be refilled every minute. Further fractionation results can be found in Supplementary Material File S1. Three fractionation qualities can be distinguished, as follows:

- No fractionation: This occurred if all the particles passed the same outlet (as can be seen in Figure 8) and can be attributed to either no particles being displaced (susp. 1 and Re = 18) or all the particles being (partially) displaced into identical outlets (susp. 1 and Re = 90).
- Type 1 fractionation: Such fractionation occurred when there was one outlet that received particles of only one size, but these particles were also found in at least one other outlet. This fractionation was considered successful as long as there was another outlet receiving particles of only the other size. This behavior was mainly caused by mixed motion or the agglomeration of particles around the posts. Partial fractionation was observed at Re = 60 for suspensions 3 and 5 and at Re = 90 for suspensions 2, 3, and 5. By shifting the outlet positions, the partial fractionation at Re 60 (susp. 5 and 3, Figure 8 and Supplementary Data S1) could be modified to an ideal fractionation.
- Type 2 (sharp) fractionation: An ideal fractionation was observed when no outlet contained particles of mixed sizes, as seen in Figure 8, with Re = 18 for suspension 5. Furthermore, an ideal fractionation was observed at Re = 60 for suspension 2.

The array was designed in such a way that the displaced particles were expected to reach the center of each outlet. However, a fanning out of the trajectories became visible, for example in Figure 8, starting at Re = 60 and increasingly at Re = 90. Consequently, the following subsections are split into two, focusing on the fully displaced particles in order to determine the critical diameter of the segments and the particles not fully displaced, moving in mixed motion.

### 3.3. Fully Displaced Particles

In order to focus on the critical diameter of the first and second segments (D_c,1_ and D_c,2_, respectively), only fully displaced particles were taken into account. Table 5 presents the predictions for the critical diameter, independent of the Reynolds number according to Equation (1), as well as the Re-dependent diameter according to Equation (2) and the particle diameters that were obtained experimentally. In Table 5, only the particles that were fully displaced within outlet 2 (O_2_) and outlet 3 (O_3_) were considered. Due to the fanning out of the particle trajectory at Re = 90, particles of a single size could be found in the center of two outlets, indicating a partial displacement in both segments, as can be seen in Figure 8 at Re = 90. As it was not possible to clearly define a single segment of displacement, particle sizes were present in multiple outlets in Table 5 at Re = 90. The range of the predicted diameters in each outlet was based on the critical diameter of each segment. For example, in the first outlet, non-displaced particles were present up to the critical diameter of the first segment (0-D_c,1_), as shown in Figure 1. Given that D_c,2_ is larger than D_c,1_, the third outlet should only contain particles larger than the second critical diameter. The gap of the array was taken as the maximal size for the third outlet as particles larger than the gap would fully block the array.

At a Re of 18 in the first segment, particles of 10 µm were displaced, while those at a smaller size were not. Particles of 8 µm partially reached the second outlet only via the mixed mode, which will be discussed in the following subsection. Furthermore, no particles were displaced by the second segment into the third outlet, which is in better agreement with Equation (1). This is at odds with the simulations, as shown in Figure 6, which predicted that particles of 10 µm would be displaced by both segments. One possible explanation for this discrepancy is that the simulations were carried out for single particles without disturbance induced by other particles. As an example, particles that become trapped in the regions of low flow velocity (dead zones), i.e., the areas in front and behind the posts, will affect the displacement motion. In the experimental investigation, only the outlet regions were recorded, and direct information about the first fractionation segment was not captured.

At Re = 60, the smallest particles that were fully displaced by Section 1 had a size of 5 µm. This is in agreement with the critical diameters predicted by Equation (2) and with the simulation of the 5 µm particles, as shown in Figure 6, which indicates displacement in the first segment. The second segment fully displaced particles of 8 µm and 10 µm, which is in agreement with the prediction of critical diameter based on Equation (2) and the simulations. As it is already known, Equation (1) is independent of the Reynolds number and thus no longer applicable.

At Re = 90, a further broadening of the particle positions was observed, as shown in Figure 8. This resulted in particles of the same size being found in more than one outlet. Thus, type 1 fractionation was achieved. By the first segment, particles larger than 2 µm were displaced, which is in agreement with predictions based on Equations (1) and (2). In the second segment, only particles of 8 µm and larger were displaced, with the exception of the experiment with a suspension containing particles of 8 µm and 10 µm. It is most likely that due to a difference in diameter of 0.2, that is, the smallest experimental size difference, inertial effects led to an increase in particle–particle interactions, which affected fractionation. In addition, inertial effects on particles are also dependent on the size, and particles of 8 µm and 10 µm may have been affected more strongly than particles with a diameter of 5 µm. However, the simulations and Equations (1) and (2) predicted the displacement of particles with a diameter of 5 µm in the second segment.

### 3.4. Mixed Motion

In this section, the focus is on particles following mixed motion, being partially displaced. In practice, the particles did not follow the zigzag motion completely but were displaced every second or third period. Consequently, these particles only reached the first few or last few micrometers of the next outlet. At low Re numbers, mixed motion is caused by geometry. Based on the simulations, the ratio of the post radius and the center-to-center distance was identified as one of the most important parameters [7]. In the experiments, mixed motion at Re = 18 was only visible in suspension 4, which contained 8 µm and 10 µm particles (see Supplementary Material File S1). Given the low difference in nominal particle sizes in this suspension (2 µm), even with a small overlap in the particle size measurements in Figure 4, this suspension was also more prone to particle–particle interactions within the array. In addition to mixed motion, at Re = 18, suspension 3, containing particles of 5 µm and 8 µm, exhibited broadened trajectories, especially for the 5 µm particles, due to the presence of a larger particle near the bifurcation between two outlets, as can be seen in Supplementary Material File S1. Due to the inability to observe the full array simultaneously, only trajectories at the outlet section could be analyzed.

At higher Re, the inertial effects increased. In the simulations, anisotropy in the flow velocity field was observed, caused by anisotropy between the inertial lift and wall force [8]. This can influence mixed motion at higher Re. Additionally, as previously established, static vortices formed behind the posts, as can be seen in Figure 8. This is also expected to influence mixed motion at higher Reynolds numbers. In the experiments, particles with a size of 2 µm had been observed to reach the second outlet by mixed motion, starting at Re = 60, a result that was unexpected. Additionally, particles with a size of 8 µm were not completely displaced by the second segment at Re = 60. In the simulations, particles with a size of 5 µm exhibited mixed motion in the second segment at Re = 60. The experiments revealed a back-focus of the particles and a further broadening of all particle sizes at Re = 90. This was evidenced by the observation that particles from all suspensions could be found in at least two outlets, as seen in Supplementary Material File S1. This indicates that at Re = 90, particles of 2 µm, 5 µm, and 8 µm were moving in mixed motion in the first segment. In the second segment, particles with a size of 10 µm and 8 µm were observed to be present in mixed motion, only partially reaching the second outlet. Suspension 3, containing particles with a size of 8 µm and 10 µm, resulted in a focus of both sizes in the second outlet, which was not anticipated. Consequently, this experiment was conducted multiple times with consistent results. Two potential explanations for this phenomenon can be proposed. First, the relatively small size difference of 2 µm and the visible overlap in Figure 5 may have led to an increase in particle–particle interactions. Second, the inertial forces upon particles may have also increased as the particle size increased. This would explain the different trajectory in this suspension compared to all the other suspensions containing either 8 µm or 10 µm particles.

### 3.5. Shift of Positions Due to the Reynolds Number

**Figure 9:**
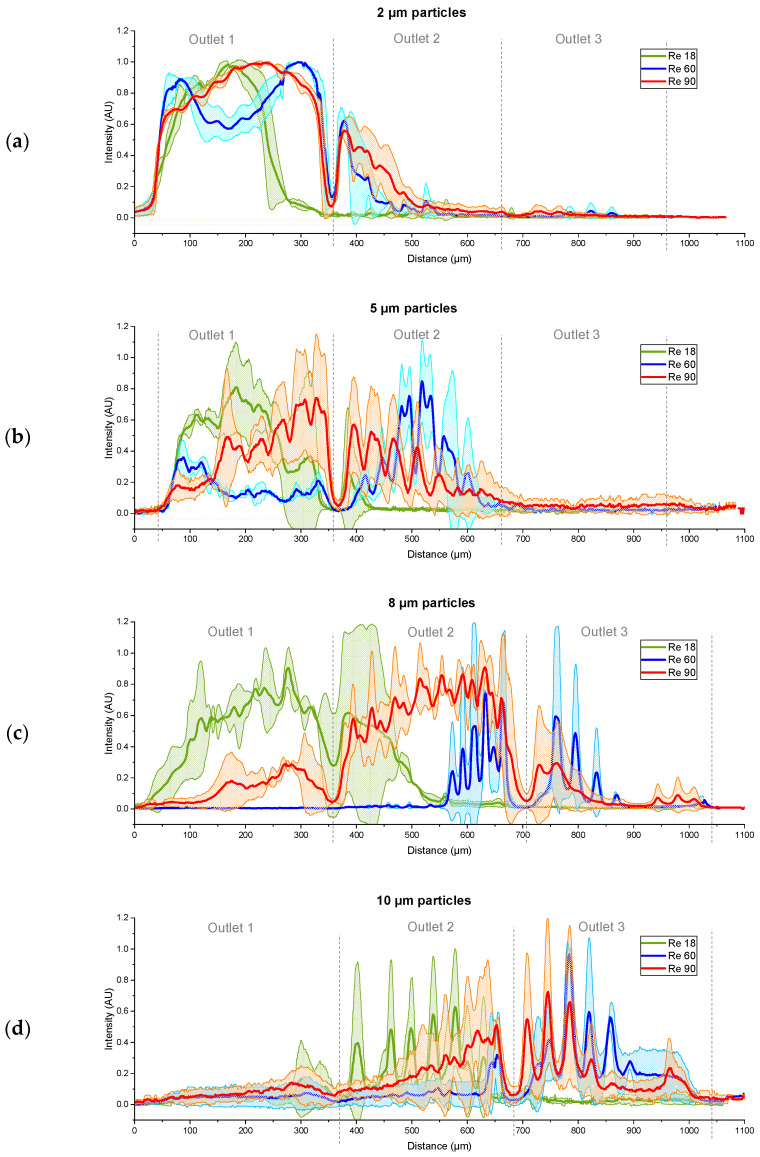
The Re-dependent shift of particle positions, obtained by averaging the intensity distributions of all the experiments in which (**a**) 2 µm, (**b**) 5 µm, (**c**) 8 µm, and (**d**) 10 µm particles were present in the bi-disperse suspensions.

**Figure 10:**
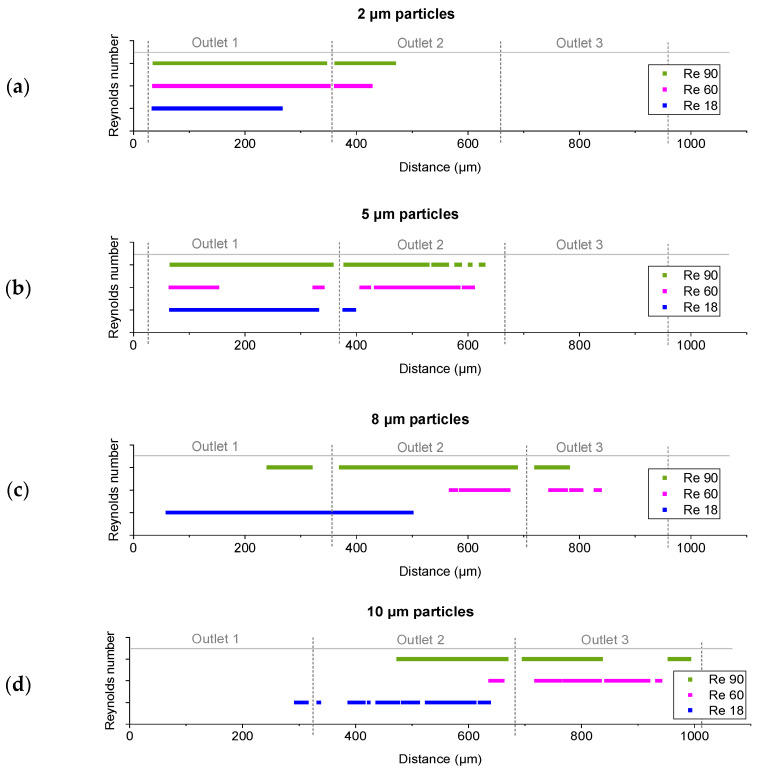
Simplified analysis of the data already shown in Figure 10. The positions where the intensities were above 20% of the maximal value of all the experiments containing (**a**) 2 µm, (**b**) 5 µm, (**c**) 8 µm, and (**d**) 10 µm particles in the bi-disperse suspensions are indicated.

To further examine the Re-dependent change in particle position, the data were rearranged to average distributions of particles of the same size, which were obtained in different experiments after referencing them to the edges of the outlet (Figure 9). To facilitate the visualization and summary of the experimental results, the data were simplified in Figure 10 by indicating the positions where the intensity values were above the threshold of 20% of the maximal value.

A Re-dependent shift of particle positions was observed across all diameters. Even particles with a diameter of 2 µm exhibited a shift in positions, resulting in mixed motion within the first segment. As shown in Figure 9a, there was even further displacement within the mixed mode of 2 µm particles at Re = 90 compared to Re = 60. For the larger particles, the shift in position occurred as expected until Re = 60. However, at Re = 90, a broadening of the positions and, as a consequence, a backshift towards the non-displaced outlet also occurred by mixed motion rather than a further increase in displacement. In addition to the increase in mixed motion, at high throughputs, there were more particles traveling through the array per time period, thereby increasing the probability of particle interactions or agglomeration around the posts. 

## 4. Conclusions

This is the first study to investigate a high-aspect-ratio DLD array in a channel with a height of 120 µm and a gap of 23 µm, addressing particles smaller than 10 µm, for an overall flow rate of up to 15 mL/min, corresponding to Re = 90, for binary fractionation. The perfect fractionation (each outlet containing only one size) of particle size combinations of 5 µm with 10 µm was obtained at Re = 18 and of 2 µm with 10 µm at Re = 60. At Re = 90, a broadening of the particle position distribution was observed, resulting in particles of the same size being found in more than one outlet. Nevertheless, suspensions containing particle combinations of 2 µm with 10 µm as well as 5 µm with 10 µm were successfully fractionated, even at the highest flow rate. At the lowest investigated flow rate of 3 mL/min, the simulations and Equation (2) predicted a displacement of 10 µm particles in both segments, which could not be confirmed experimentally. However, Equation (1) predicted that at these conditions, 10 µm particles would only be displaced in the first segment. The simulations were conducted for single particles traversing each segment. It should be noted that particle–particle interactions and stuck particles were not considered in the simulations. At Re = 60, the simulations were in good agreement with the experiments, although a fraction of 5 µm particles was observed in the second outlet. At Re = 90, the fanning out of the particle trajectory was observed in the experiments but not in the simulations. It can be assumed that this discrepancy was a result of particle–particle interactions, including those with stuck particles that occurred in the experimental process. The arrays of this study exhibited mixed motion that was likely advanced by the inertial wall and lift forces at higher Reynolds numbers as well as by particle agglomeration around the posts (which is difficult to completely avoid in DLD). This study indicates that higher aspect ratios are highly effective for increasing throughput in DLD devices and do not reduce selectivity. Since the maximal pressure was 15 bar in the investigated arrays, it is necessary to take the stability of the posts into account when increasing the aspect ratio further. Increasing the throughput by higher flow velocities leads to a decrease in selectivity when Re = 60 is exceeded, which may not be acceptable for all fractionation tasks. The unprecedented total fractionation throughput of almost 1 L per hour demonstrated here, which was made possible in a pressure-tight DLD device with a high aspect ratio, opens up avenues for completely new applications for chemical, pharmaceutical, and recycling technologies.

## Figures and Tables

**Table 1:** Volumetric flow rates through the buffer and sample inlets.

Overall Flow Rate	Re	Buffer 1	Sample	Displacement Buffer
3 mL/min	18	0.35 mL/min	0.35 mL/min	2.3 mL/min
10 mL/min	60	1.2 mL/min	1.2 mL/min	7.6 mL/min
15 mL/min	90	1.8 mL/min	1.8 mL/min	11.4 mL/min

**Table 2:** Overview of the different parameter combinations in the CFD-DEM simulations.

Inlet Velocity	Reynolds Number (Re)	Segment	Post Rows	Tilt Angle (*θ*)	Particle Diameter (*D*)	Domain Depth
0.463 m/s	18	1	21	2.7°	5 µm	10 µm
					10 µm	20 µm
		2	8	7.1°	5 µm	10 µm
					10 µm	20 µm
1.545 m/s	60	1	21	2.7°	5 µm	10 µm
					10 µm	20 µm
		2	8	7.1°	5 µm	10 µm
					10 µm	20 µm
2.318 m/s	90	1	21	2.7°	5 µm	10 µm
					10 µm	20 µm
		2	8	7.1°	5 µm	10 µm
					10 µm	20 µm

**Table 3:** Summary of the selected DEM parameters.

Description	Value	Unit
Coefficient of restitution	0.1	(-)
Poisson ratio	0.5	(-)
Static friction coefficient	0.1	(-)
Density of particle	1000	kg/m^3^
Young’s modulus	3 × 10^8^	Pa
Surface roughness	0.05 × r_particle_	m
Activation distance for lubrication correction	0.4058 × 10^−6^	m
Density of fluid	1000	kg/m^3^
Kinematic viscosity	1 × 10^−6^	m^2^/s

**Table 4:** Overview of the binary particle suspensions that were used in the experiments at varied Reynolds numbers.

Test Suspension	Green Fluorescent	Red Fluorescent	Re
1	2 µm	5 µm	18 60 90
2	2 µm	10 µm	
3	8 µm	5 µm	
4	8 µm	10 µm	
5	10 µm	5 µm	

**Table 5:** Experimentally observed fully displaced particle sizes found in outlets O_1_, O_2_, and O_3_, together with predicted size ranges according to Equations (1) and (2). All the particle sizes that were found in certain outlets only at very small fractions caused by mixed motion are not given here.

	O_1_ (exp.)	O_1_ (pred.)	O_2_ (exp.)	O_2_ (pred.)	O_3_ (exp.)	O_3_ (pred.)
Equation (1)		0–7.8 µm		7.8–11.8 µm		11.8–23 µm
Re 18	2 µm 5 µm 8 µm	0–5.6 µm	10 µm	5.6–8.4 µm	-	8.4–23 µm
Re 60	2 µm	0–3.7 µm	5 µm 8 µm	3.7–5.5 µm	8 µm ^1^ 10 µm	5.5–23 µm
Re 90	2 µm 5 µm 8 µm	0–2.7 µm	5 µm 8 µm 10 µm	2.7–4.1 µm	8 µm 10 µm	4.1–23 µm

^1^ Only for suspension 3 containing particles with a size of 5 µm and 8 µm.

## Data Availability

The data that support the findings of this study are available on request from the corresponding author.

## Supplementary Materials

The following supporting information can be downloaded at: https://www.mdpi.com/article/10.3390/mi15060802/s1, Supplementary File S1: Full-particle fractionation profiles.
