# Supplementary material for: A High-Aspect-Ratio Deterministic Lateral Displacement Array for High-Throughput Fractionation
Source: Micromachines (Basel). 2024 Jun 18;15(6):802. doi: 10.3390/mi15060802 (PMC11205752; doi:10.3390/mi15060802)
Supplement: Supplementary file 1 [file micromachines-15-00802-s001.zip › micromachines-2996934-supplementary.pdf]

Supplementary data File S1

Supplementary information 1: Fabrication process

Table S1: Illustrated process plan

1	Array structuring	
1a	Adhesion promotion	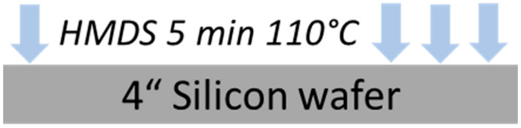
1b	Resist coating<br><br>*Softbake (5 min, 110°C) not depicted*	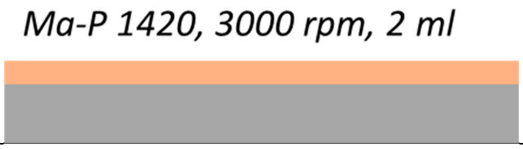
1c	Mask alignment and exposure	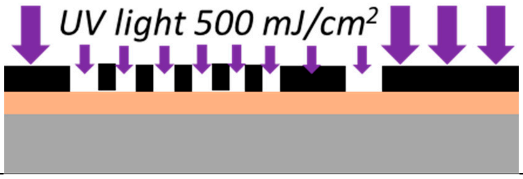
1d	Development<br><br>*Hardbake (10 min, 110°C) not depicted*	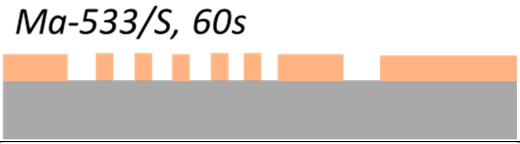
1e	Deep reactive ion etching	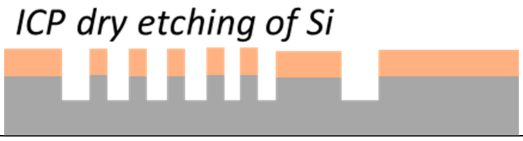
1f	Ashing of the resist in ICP<br>Wafer cleaning	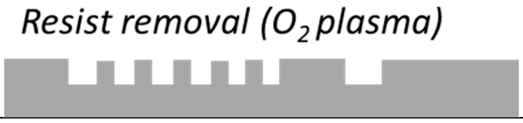
2	Backside structuring	
2a	Resist coating<br><br>*Softbake (1 min, 110°C) not depicted*	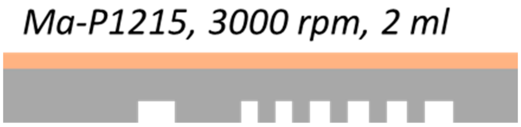
2b	Development<br><br>*Hardbake (5 min, 110°C) not depicted*	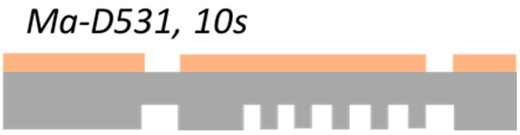
2c	Array protection<br><br>*Hardbake (20 min, 110°C) not depicted*	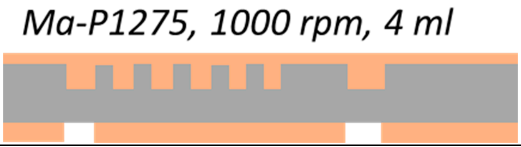

2d	Structuring the fluidic vias<br><br>*By fs-laser ablation*	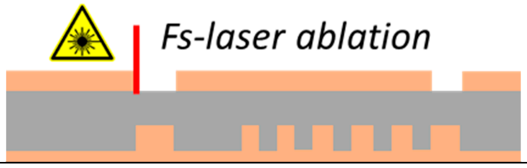
2e	Resist removal and wafer cleaning<br><br>*Removal by acetone, cleaning by H_2SO_4:H_2O_2 (2:1)*	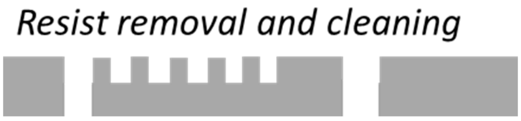
3	Oxide layer	
	Thermal oxide 300 nm	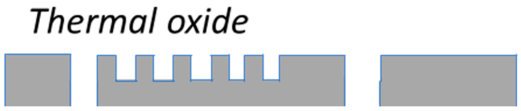
4	Wafer bonding	
	Anodic bonding<br><br>*Wafer and cover cleaning by H_2SO_4:H_2O_2 (2:1) not depicted*<br>Cover: 0.5 mm borosilicate glass	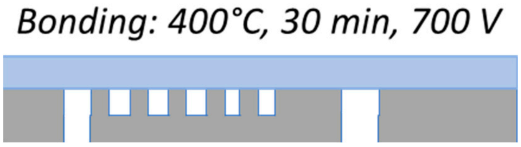
5	Dicing not depicted	

Table S2: Process parameters for dry etching of the DLD arrays resulting in a height of 120 µm. The temperature was constant in all steps at 5 °C. Gas flows are given in sccm (standard cubic centimeter per minute).

Parameter	Deposition step	Breakthrough step	Etch step
APC (chamber pressure)	35 mTorr (4.66 Pa)	30 mTorr (3.99 Pa)	40 mTorr (5.33 Pa)
ICP RF	1500 W	1300 W	1500 W
Table Power RF	5 W	45 W	0 W
SF6	10 sccm	200 sccm	200 sccm
C4F8	180 sccm	10 sccm	10 sccm
Time	2.3 s	2.1 s	2.7 s

Supplementary information 2: Experimental setup

The schematic experimental setup consisting of syringe pumps, holder with the system attached and the microscope for observation is depicted in Figure S1a. An actual photo of the setup with the different sections labeled is depicted in Figure S1b. Due to the high pressures involved, stainless steel syringes attached to three nemesys mid-pressure modules were used. For powering the modules, a nemesys base 120 module was used. The control of the syringes was done using the QMix elements software from Cetoni.

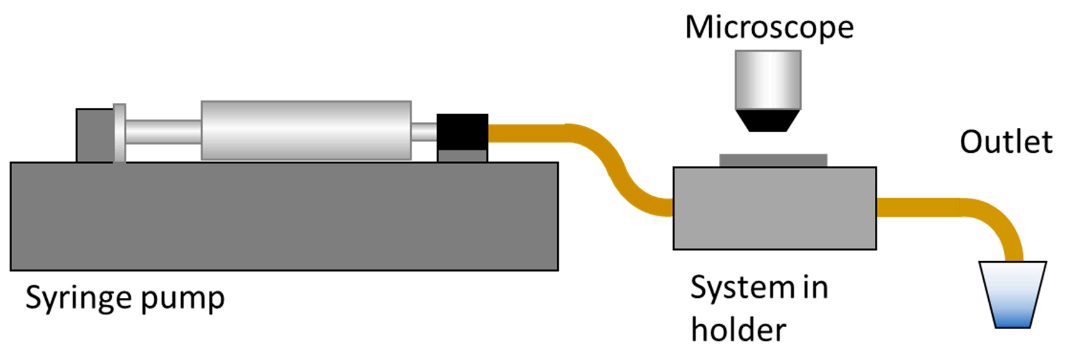

S1(**a**) Schematic view

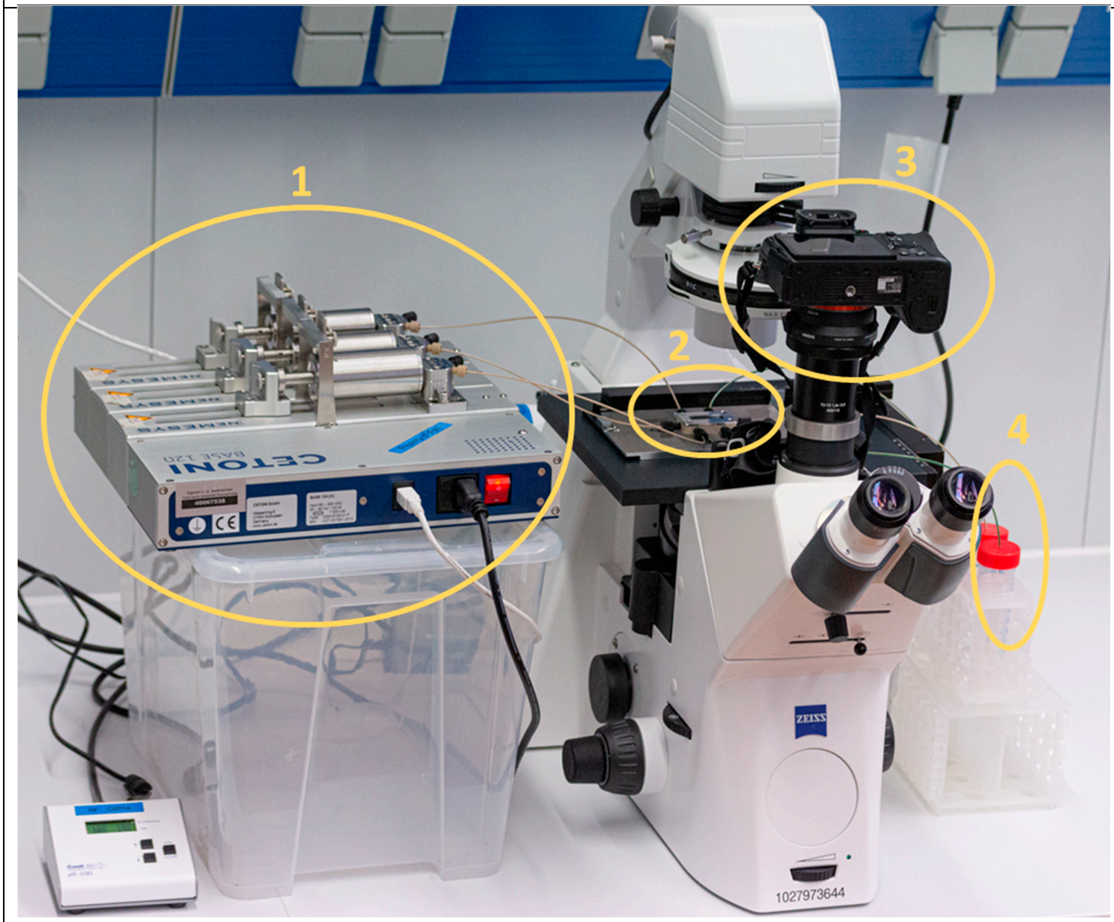

S1(**b**) Photograph of the experimental setup: 1-syringe pumps; 2-System in holder; 3-Camera; 4-Waste. The PC to control the setup is not depicted.

Supplementary information 3: full fractionation profiles

Figure S2: Full fractionation profiles of all experiments.

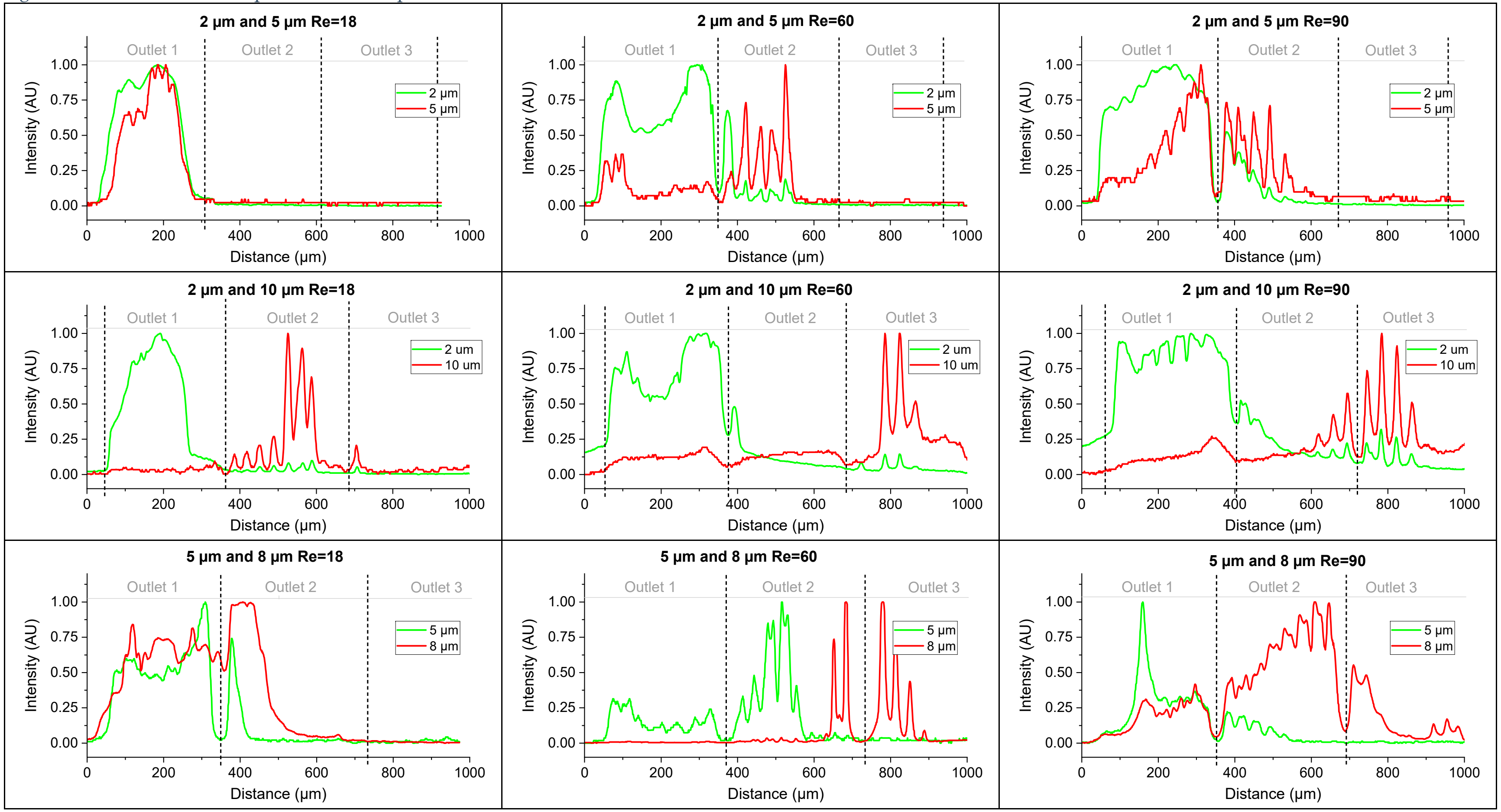

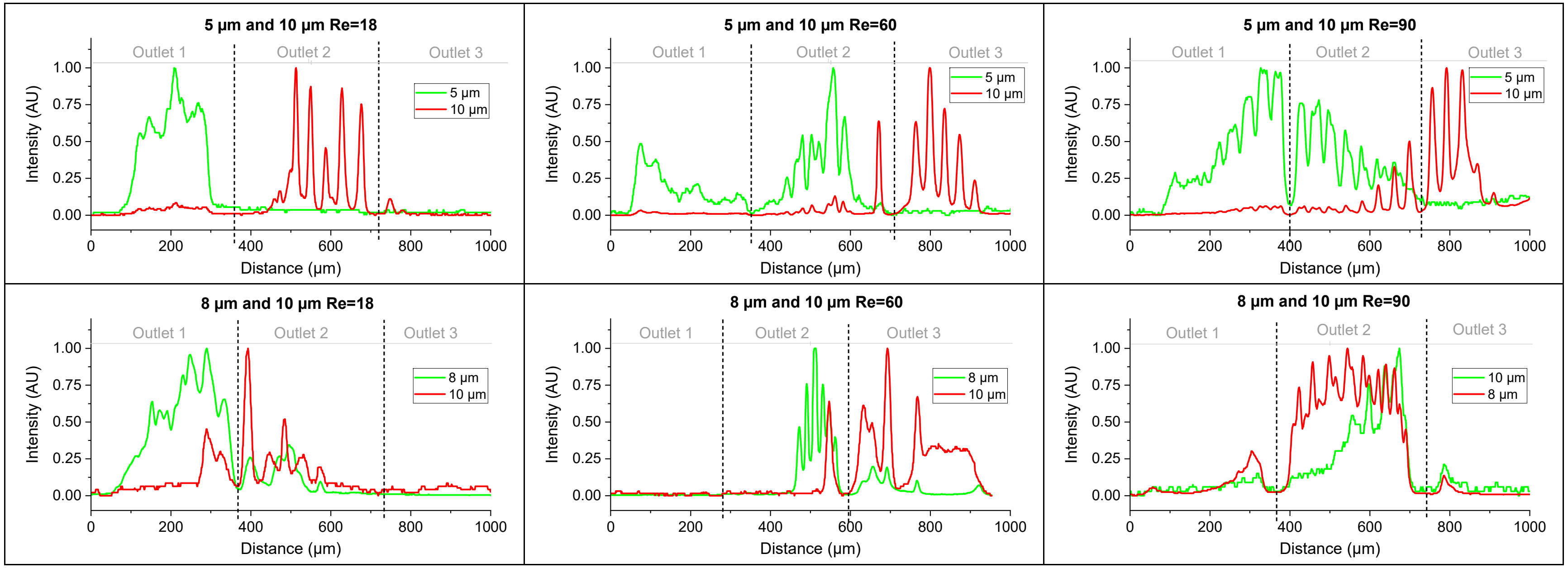
5 µm and 10 µm Re=18
5 µm and 10 µm Re=60
5 µm and 10 µm Re=90
8 µm and 10 µm Re=18
8 µm and 10 µm Re=60
8 µm and 10 µm Re=90
Outlet 1
Outlet 2
Outlet 3
5 µm
10 µm
8 µm
Intensity (AU)
Distance (µm)
0.00
0.25
0.50
0.75
1.00
0
200
400
600
800
1000
